# MSCs-engineered biomimetic PMAA nanomedicines for multiple bioimaging-guided and photothermal-enhanced radiotherapy of NSCLC

**DOI:** 10.1186/s12951-021-00823-6

**Published:** 2021-03-20

**Authors:** Yipengchen Yin, Yongjing Li, Sheng Wang, Ziliang Dong, Chao Liang, Jiaxin Sun, Changchun Wang, Rong Chai, Weiwei Fei, Jianping Zhang, Ming Qi, Liangzhu Feng, Qin Zhang

**Affiliations:** 1 Department of Radiation Oncology, Shanghai Chest Hospital, Shanghai Jiao Tong University, Shanghai, 200030 P. R. China; 2 State Key Laboratory of Molecular Engineering of Polymers, Department of Macromolecular Science, and Laboratory of Advanced Materials, Fudan University, Shanghai, 200438 P. R. China; 3 Department of Colorectal Surgery, Fudan University Shanghai Cancer Center, Shanghai, 200032 P. R. China; 4 Jiangsu Key Laboratory for Carbon-Based Functional Materials & Devices, Institute of Functional Nano & Soft Materials (FUNSOM), Soochow University, Suzhou, 215123 Jiangsu P. R. China; 5 Department of Nuclear Medicine, Fudan University Shanghai Cancer Center, Shanghai, 200032 P. R. China; 6 Key Laboratory of Nuclear Physics and Ion-Beam Application (MOE), Fudan University, Shanghai, 200433 P. R. China

**Keywords:** Cancer theranostics, Biomimetic nanoparticles, Active targeting, Bimodal imaging, Photothermal therapy, Radiotherapy, Non-small cell lung cancer

## Abstract

**Background:**

The recently developed biomimetic strategy is one of the mostly effective strategies for improving the theranostic efficacy of diverse nanomedicines, because nanoparticles coated with cell membranes can disguise as “self”, evade the surveillance of the immune system, and accumulate to the tumor sites actively.

**Results:**

Herein, we utilized mesenchymal stem cell memabranes (MSCs) to coat polymethacrylic acid (PMAA) nanoparticles loaded with Fe(III) and cypate—an derivative of indocyanine green to fabricate Cyp-PMAA-Fe@MSCs, which featured high stability, desirable tumor-accumulation and intriguing photothermal conversion efficiency both in vitro and in vivo for the treatment of lung cancer. After intravenous administration of Cyp-PMAA-Fe@MSCs and Cyp-PMAA-Fe@RBCs (RBCs, red blood cell membranes) separately into tumor-bearing mice, the fluorescence signal in the MSCs group was 21% stronger than that in the RBCs group at the tumor sites in an in vivo fluorescence imaging system. Correspondingly, the *T*_1_-weighted magnetic resonance imaging (MRI) signal at the tumor site decreased 30% after intravenous injection of Cyp-PMAA-Fe@MSCs. Importantly, the constructed Cyp-PMAA-Fe@MSCs exhibited strong photothermal hyperthermia effect both in vitro and in vivo when exposed to 808 nm laser irradiation, thus it could be used for photothermal therapy. Furthermore, tumors on mice treated with phototermal therapy and radiotherapy shrank 32% more than those treated with only radiotherapy.

**Conclusions:**

These results proved that Cyp-PMAA-Fe@MSCs could realize fluorescence/MRI bimodal imaging, while be used in phototermal-therapy-enhanced radiotherapy, providing desirable nanoplatforms for tumor diagnosis and precise treatment of non-small cell lung cancer.

**Supplementary Information:**

The online version contains supplementary material available at 10.1186/s12951-021-00823-6.

## Background

Non-small cell lung cancer (NSCLC) is one of the leading causes of death worldwide, against which radiotherapy (RT) is commonly used for NSCLC treatment [1–3]. However, the overall survival rate in NSCLC patients treated with RT is still far from satisfactory owing to the RT resistance, RT-related/induced adverse effects, etc. [4, 5]. Photothermal therapy (PTT) is an emerging treatment modality which utilizes photothermal nanoagents to convert near-infrared (NIR) light energy into thermal energy for effective tumor ablation. It can induce vasodilation of tumor to reduce tumor hypoxia, thereby reducing RT resistance and improving the RT efficacy [6–18]. Nanoparticles with the nanoscale size can accumulate into the tumor site, which can be developed as contrast agents of bioimaging to be applied in multiple bioimaging systems such as fluorescence imaging system, magnetic resonance imaging (MRI) system, etc., which can either enable or assist the early diagnosis and precise treatment of cancer [19–23].

Bone marrow mesenchymal stem cell (MSC) is one kind of the stem cells, which features the potential to differentiate into various types of connective tissues, and shows promising application prospects in tissue engineering such as burns, degenerative diseases, and cell replacement therapy of cancer [24–34]. MSC is of low immunogenicity, therefore nanoparticles coated with MSCs can disguise as “self”, thereby reducing the phagocytosis of reticular endothelial system, evading the surveillance of the immune system, prolonging the circulation time in vivo, and accumulating at the tumor site through the enhanced permeability and retention effect [35, 36]. More importantly, MSC is capable of inflammation aggregation and tumor-active targeting through intercellular interactions and chemokines [37]. Therefore, MSCs-coated nanoparticles can target to tumors more actively and efficiently compared with erythrocyte-membranes-coated nanoparticles. However, a quantitative comparison of tumor-targeting capability to MSCs-coated nanoparticles v.s. erythrocyte-membranes-coated nanoparticles has not been reported yet. In addition, MSC has rich sources and is easy to culture, proliferate, being isolated and purified, which provides convenience for its practical clinical translation and applications [38].

**Scheme 1:**
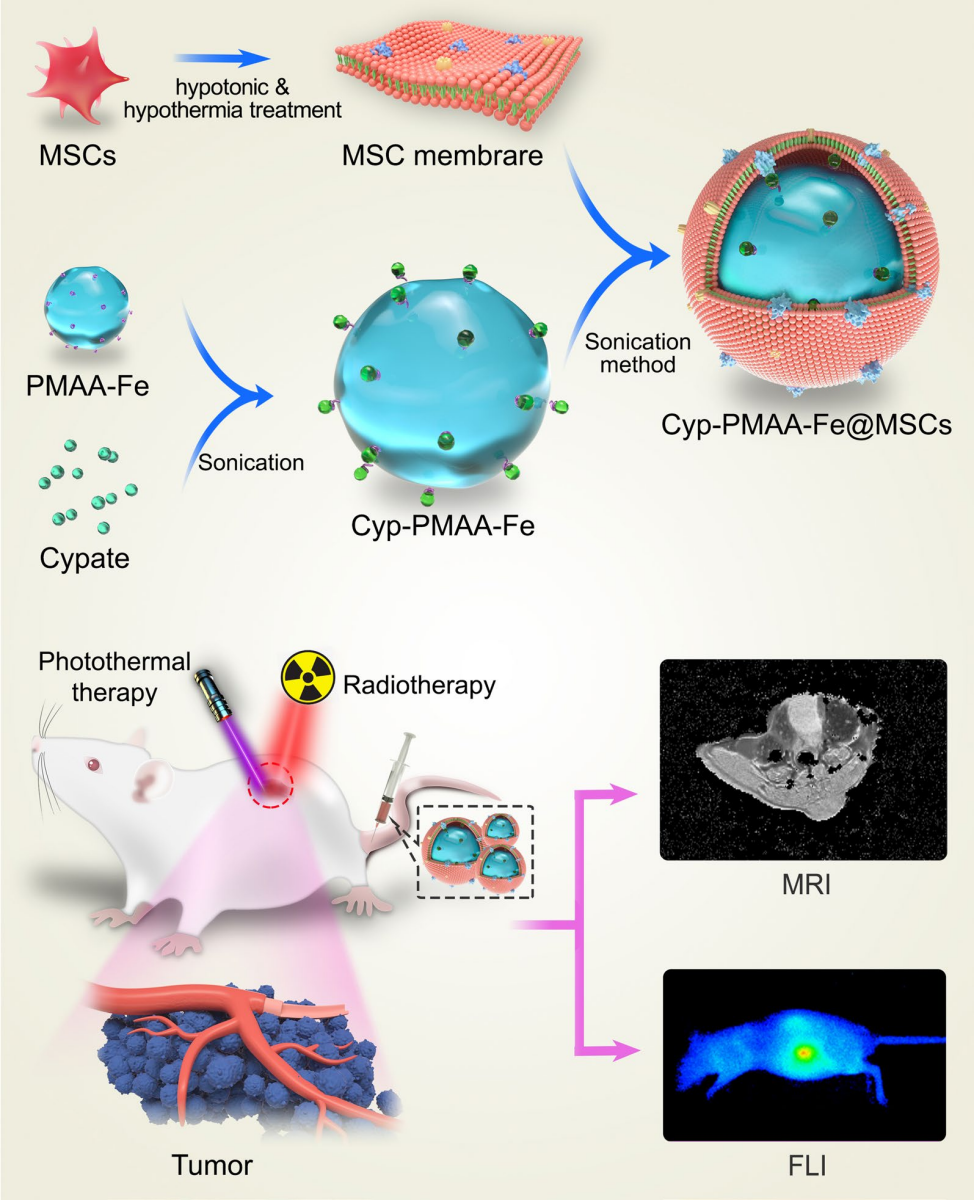
Schematic illustration of the construction of Cyp-PMAA-Fe@MSCs nanomedicines for fluorescence imaging and MR imaging-guided photothermal-enhanced radiotherapy of NSCLC

In this work, we used the reflux precipitation polymerization approach to construct soft organic nanoparticles polymethacrylic acid (PMAA), which were featured with low toxicity, swelling behavior and distinct deformability (Scheme 1) [39–42]. When loaded with Fe(III), PMAA nanoparticles could be employed for *T*_1_-weighted MRI. After intravenous injection of nanoparticles in tumor-bearing mice, the *T*_1_-weighted MRI signal was decreased 30% at the tumor site, indicating that they acted as contrast agents for *T*_1_-weighted MRI. Cypate was a derivative of indocyanine green with bis-carboxyl groups (Additional file 1: Figure S1), which was capable of emitting fluorescence, generating heat under NIR irradiation [43]. Cypate could coordinate with Fe(III) and be combined with PMAA nanoparticles, and then be coated with MSCs to fabricate Cyp-PMAA-Fe@MSCs nanomedicines, which exhibited high stability and photothermal-conversion efficiency. After intravenous administration of Cyp-PMAA-Fe@MSCs and Cyp-PMAA-Fe@RBCs (RBCs, red blood cell membranes) separately in tumor-bearing mice, Cyp-PMAA-Fe@MSCs group had a 21% stronger fluorescence signal at the tumor site than Cyp-PMAA-Fe@RBCs group, indicating that Cyp-PMAA-Fe@MSCs accumulated more at the tumor site. Furthermore, mice were treated with Cyp-PMAA-Fe@MSCs for synergistic PTT/RT. The therapeutic effect was 32% better than mice treated with RT alone. Therefore, the engineered Cyp-PMAA-Fe@MSCs nanomedicine was an intriguing nanoplatform with the characteristic of low toxicity, high stability, and active tumor-targeting efficiency. It could be used for not only fluorescence/*T*_1_-weighted MRI bimodal imaging, but also PTT under NIR irradiation to improve the therapeutic effect of NSCLC RT treatment.

## Results and discussion

**Fig. 1:**
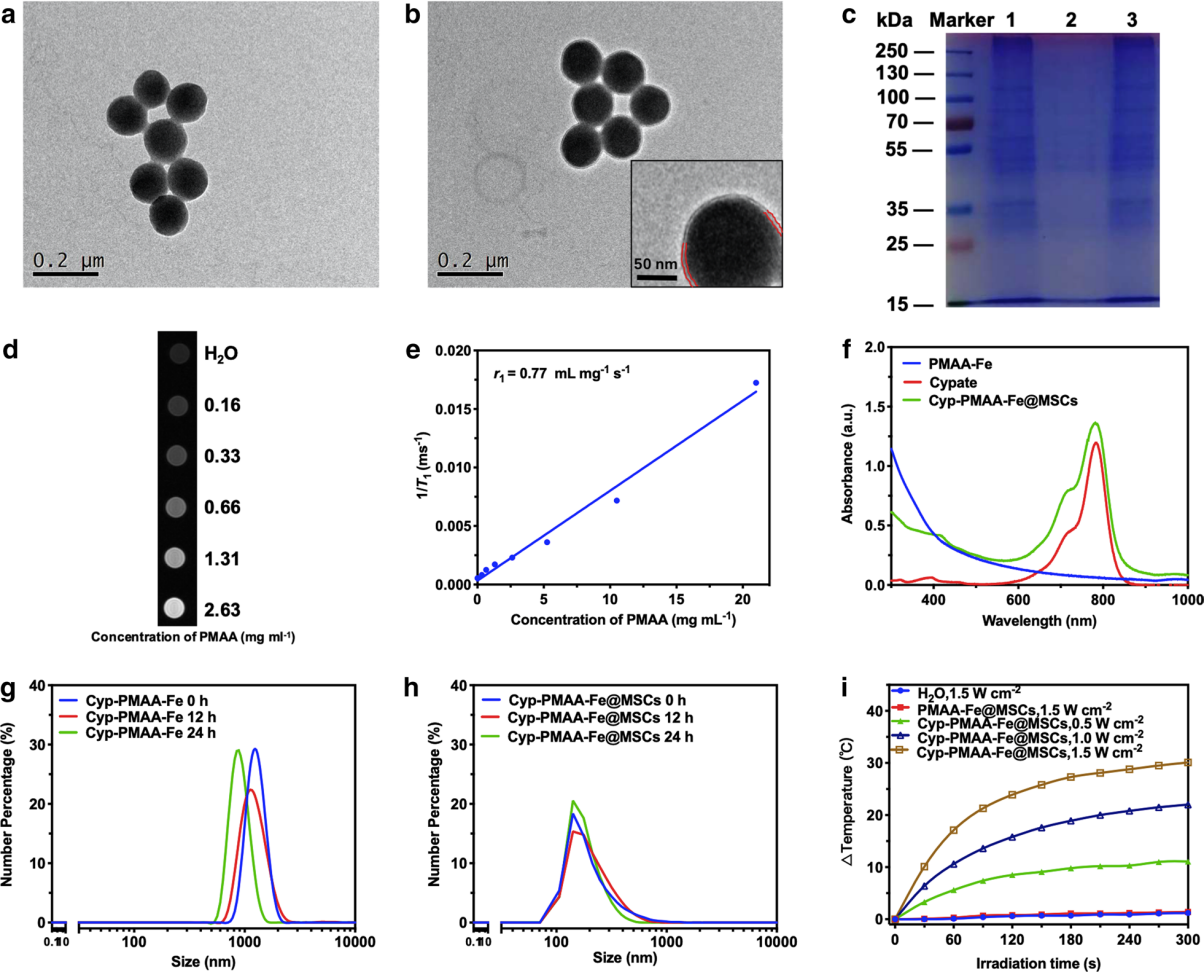
Preparation and characterization of PMAA-Fe and Cyp-PMAA-Fe@MSCs. **a** TEM image of PMAA-Fe. **b** TEM image of Cyp-PMAA-Fe@MSCs. Inset: A representative high-resolution TEM (HRTEM) image. **c **Protein bands analysis of MSCs, Cyp-PMAA-Fe, and Cyp-PMAA-Fe@MSCs. Samples were run on polyacrylamide gel electrophoresis at equivalent concentration and stained with Coomassie Brilliant Blue. **d** *T*_1_-weighted MR images of Cyp-PMAA-Fe@MSCs solutions at different PMAA concentrations. **e** *T*_1_ relaxation rate (*r*_1_) of Cyp-PMAA-Fe@MSCs solutions measured at different PMAA concentrations. **f** Ultraviolet–visible-NIR absorbance spectra of cypate dispersed in methanol, as well as PMAA-Fe and Cyp-PMAA-Fe@MSCs dispersed in DI water. (**g**, **h**) Dynamic Light Scattering distribution of Cyp-PMAA-Fe (G) and Cyp-PMAA-Fe@MSCs (**h**) in PBS. (**i**) Photothermal heating curves of Cyp-PMAA-Fe@MSCs as well as DI water and PMAA-Fe@MSCs irradiated under 808 nm laser for 5 min with different laser power densities

PMAA nanoparticles were constructed with reflux-precipitation polymerization approach according to previous study [44]. MSCs were derived from cultured MSC and seperated via hypotonic and centrifugation method. The PMAA nanoparticles exhibited spherical morphology with uniform sizes and spherical shapes by transmission electron microscope (TEM) observation (Fig. 1a). The shape of Cyp-PMAA-Fe@MSCs was basically the same as that of PMAA. The cores were composed of PMAA nanospheres, and transparent circles could be observed around the nanospheres (Fig. 1b). The thickness of the translucent circles was about 10 nm. By comparison, it could be deduced that the circles surrounding the nanospheres were MSCs, while the thickness of a single-layer MSCs was about 7 nm-15 nm, which coincided with the TEM results. The sodium dodecylsulfate polyacrylamide gel electrophoresis (SDS-PAGE) analysis showed that the protein pattern of MSCs was the same as that of Cyp-PMAA-Fe@MSCs (Fig. 1c).

The content of Fe(III) in PMAA nanoparticles was 13.3% of the total mass, which was detected by inductively coupled plasma atomic-emission spectroscopy (ICP-AES). The high content of Fe(III) ensured that PMAA nanoparticles could load sufficient amount of Cypate for PTT and MRI. As the concentration of PMAA increased, its *T*_1_-weighted MRI signal gradually increased as well. Within a certain range, the *T*_1_-weighted MRI signal intensity was linearly related to the PMAA con centration, and the longitudinal relaxation rate *r*_1_ = 0.77 mL mg^−1^ s^−1^ (Fig. 1d, e).

Cypate was coordinated with iron through complexation reaction to prepare Cyp-PMAA-Fe@MSCs, which was analyzed under ultraviolet–visible spectrophotometer in the range of 300 nm–1000 nm. The absorption peak of PMAA was at 300 nm and Cypate was at 785 nm. The absorption spectrum of Cyp-PMAA-Fe@MSCs was the overlap of the above two, and there were two absorption peaks at 300 nm and 785 nm, respectively (Fig. 1f). Further analysis revealed that the drug-loading efficiency was 21.47% and the encapsulation rate was 91.15%.

The average hydrodynamic diameter and zeta potential of PMAA-Fe were 210.8 nm and − 22.7 mV, respectively. The average hydrodynamic diameter and zeta potential of Cyp-PMAA-Fe@MSCs were 248.4 nm and − 22.3 mV, respectively (Additional file 1: Figure S2). The hydrodynamic diameter of the nanoparticles increased by 37.6 nm after coated with MSCs, while the thickness of the single-layer MSC cell membrane was 7 nm–15 nm. The increase in the particle size of the nanoparticles was close to the thickness of the double-layer MSCs. The zeta potential of Cyp-PMAA-Fe@MSCs (− 22.3 mV) was close to the zeta potential of MSCs (− 22.8 mV), which could prove that Cyp-PMAA-Fe@MSCs nanoparticles were coated with MSCs (Additional file 1: Figure S2). Cyp-PMAA-Fe formed visible sediment with an average hydrodynamic diameter above 1000 nm (Fig. 1g). After being coated with MSCs, the hydrodynamic diameter of Cyp-PMAA-Fe@MSCs did not increase significantly within 24 h, and the hydrodynamic diameter kept around 200 nm (Fig. 1h), indicating that the membrane-coating substaintially enhanced the stability of Cyp-PMAA-Fe@MSCs nanoparticles. Water, PMAA, and Cyp-PMAA-Fe@MSCs were irradiated by an 808 nm NIR laser with the power density of 1.5 W cm^−2^ for 5 min, and the temperature rose by 1.2 °C, 1.4 °C, and 30.1 °C, respectively. Cyp-PMAA-Fe@MSCs was irradiated with an 808 nm NIR laser with the power density of 0.5, 1.0, and 1.5 W cm^−2^ for 5 min, and the temperature rose by 11.1 °C, 22.0 °C, and 30.1 °C, respectively, indicating that higher power density of NIR laser induced stronger photothermal effect (Fig. 1I). Moreover, according to previous reported methods [45], the photothermal conversion efficiency of Cyp-PMAA-Fe@MSCs was calculated to be about 47.2% (Additional file 1: Figure S6).

**Fig. 2:**
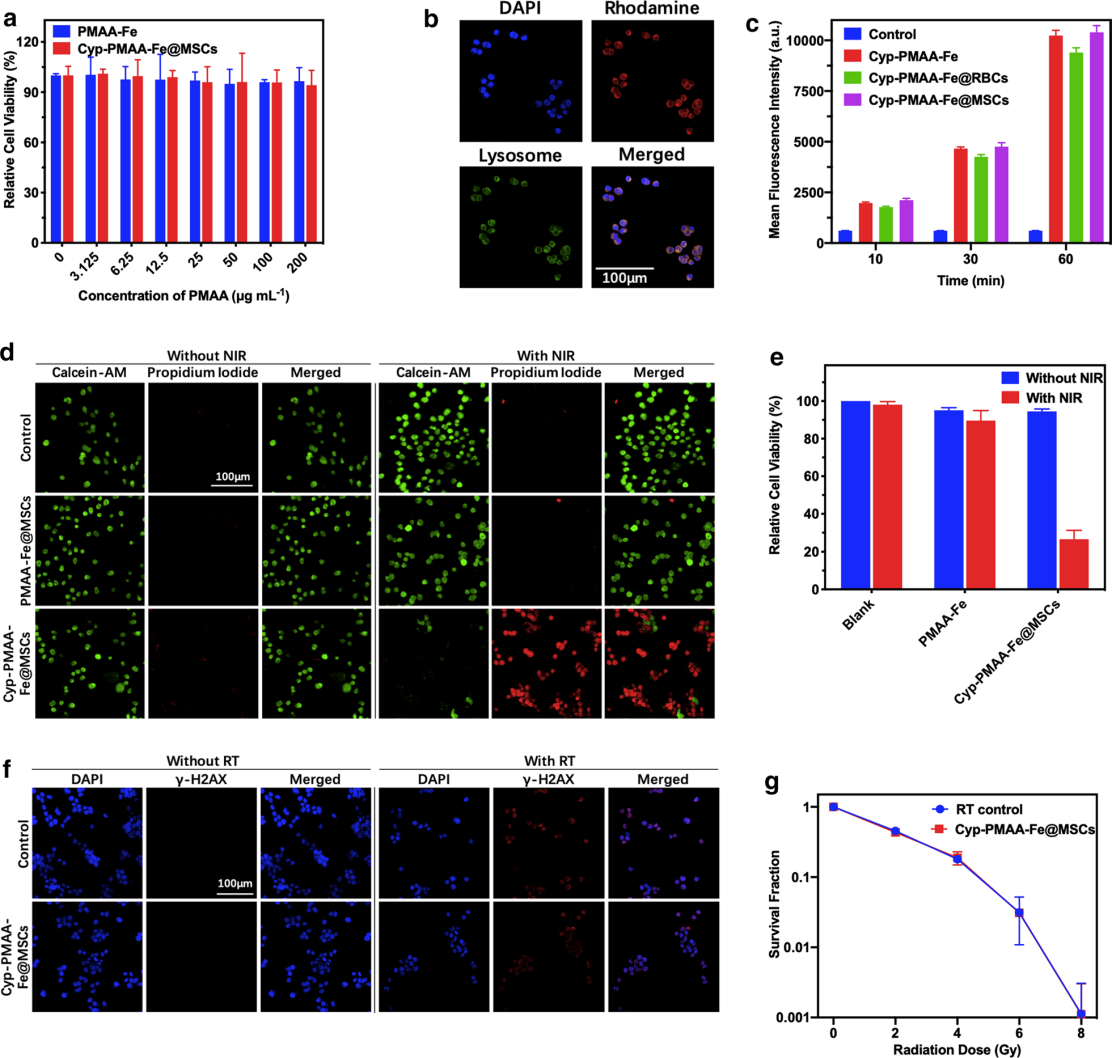
In vitro cell experiments. **a** Relative cell viabilities of LLC1 cells incubated in PMAA-Fe and Cyp-PMAA-Fe@MSCs with different concentrations for 24 h. **b** Confocal microscopic images of LLC1 cells incubated with Rhodamine-B-labeled PMAA-Fe@MSCs (red). Cell nucleus were dyed with DAPI (blue) and lysosomes were dyed with Lyso-tracker (green). **c** Flow cytometer data of LLC1 cells incubated in DMEM, Cyp-PMAA-Fe, Cyp-PMAA-Fe@RBCs and Cyp-PMAA-Fe@MSCs for 10, 30 and 60 min. **d** Confocal microscopic images of LLC1 cells treated with PBS, PMAA-Fe@MSCs and Cyp-PMAA-Fe@MSCs with/without 808 nm laser. Live and dead cells were dyed with Calcein-AM (green) and propidium iodide (red), respectively. **e** Relative cell viabilities of LLC1 cells incubated in DMEM, PMAA-Fe and Cyp-PMAA-Fe@MSCs with/without 808 nm laser. **f** Confocal microscopic images of marker of DNA breaks (γ-H2AX) in LLC1 cells treated with PMAA-Fe@MSCs with/without X- ray. **g** Clonogenic survival assay of LLC1 cells incubated with Cyp-PMAA-Fe@MSCs under different radiation doses

The cytotoxicity of PMAA and Cyp-PMAA-Fe@MSCs was assessed by Cell Counting Kit-8 (CCK-8) test. Even if the concentration of PMAA was as high as 200 μg mL^−1^, the relative cell viability is 96.59% when adding PMAA. The relative viability was 94.18% when adding Cyp-PMAA-Fe@MSCs (PMAA = 200 μg mL^−1^). The low toxicity of Cyp-PMAA-Fe@MSCs indicated the high biocompatibility of the constructed nanomedicines (Fig. 2a).

PMAA-Fe@MSCs nanoparticles labeled with the red fluorescence dye rhodamine-B (PMAA = 200 μg mL^−1^, rhodamine B = 10 μg mL^−1^) showed red fluorescence under confocal microscopy. Most of them were distributed in the lysosome and the cytoplasm where the lysosome is located, and a small number of them were distributed in the nucleus (Fig. 2b), indicating that the nanoparticles had been engulfed by LLC1 cells and the nanoparticles could be enriched in LLC1 cells. In the flow cytometry assay, the signal in Cyp-PMAA-Fe@MSCs group was higher than Cyp-PMAA-Fe@RBCs group at every time point (Fig. 2c), indicating that when coated with MSCs, the nanoparticles were more easily to be uptaken by LLC1 cells.

In order to evaluate the photothermal treatment ability of Cyp-PMAA-Fe@MSCs objectively, the LLC1 cells were stained by Calcein-AM (live cells) and propidium iodide (dead cells). When the concentration of Cyp-PMAA-Fe@MSCs was 1 mg mL^−1^ and the irradiation power density was 1.5 W cm^−2^, almost all the LLC1 cells died and were stained red by propidium iodide. When PMAA (PMAA = 200 μg mL^−1^) was added, the irradiation conditions were the same as before. There was almost no cell death and green fluorescence displayed almost in the full field of view (Fig. 2d). For the blank group, PMAA group, and Cyp-PMAA-Fe@MSCs group (PMAA = 200 μg mL^−1^), the relative cell viabilities of LLC1 cells were 100%, 95.07%, and 94.44%, respectively, after 5 min of NIR irradiation (irradiation power = 0 W cm^−2^). When the power density of 808 nm NIR laser rose to 1.5 W cm^−2^, the relative cell viabilities of LLC1 cells were 98.09%, 89.54% and 26.48%, respectively (Fig. 2e). The relative cell viabilities of LLC1 cells in Cyp-PMAA-Fe@MSCs with laser irradiation group is 3.57 times higher than that of without laser irradiation group. The results demonstrated that Cyp-PMAA-Fe@MSCs featured a high photothermal treatment performance.

γ-H2AX labeled with red fluorescence dye Cy3 showed the fracture of double-stranded deoxyribonucleic acid (DNA), and the LLC1 cells irradiated with X-rays exhibited red fluorescence under confocal microscopy, indicating the occurrence of DNA damage. Whether Cyp-PMAA-Fe@MSCs (PMAA = 200 μg mL^−1^) was added or not, there was no DNA damage in the absence of X-ray exposure. DNA damage occurred in the presence of X-ray exposure, and the degree of DNA damage was similar between the nanomedicine-added group and the nanomedicine-non-added group (Fig. 2f). In clonogenic assay, the surviving fraction of LLC-1 cells was decreased with the elevation of X-ray dose, while the surviving fraction was close between with Cyp-PMAA-Fe@MSCs group (PMAA = 200 μg mL^−1^) and without Cyp-PMAA-Fe@MSCs group (Fig. 2g), indicating that the introduced nanoparticles did not affect the absorption of X-ray of LLC1 cells.

**Fig. 3:**
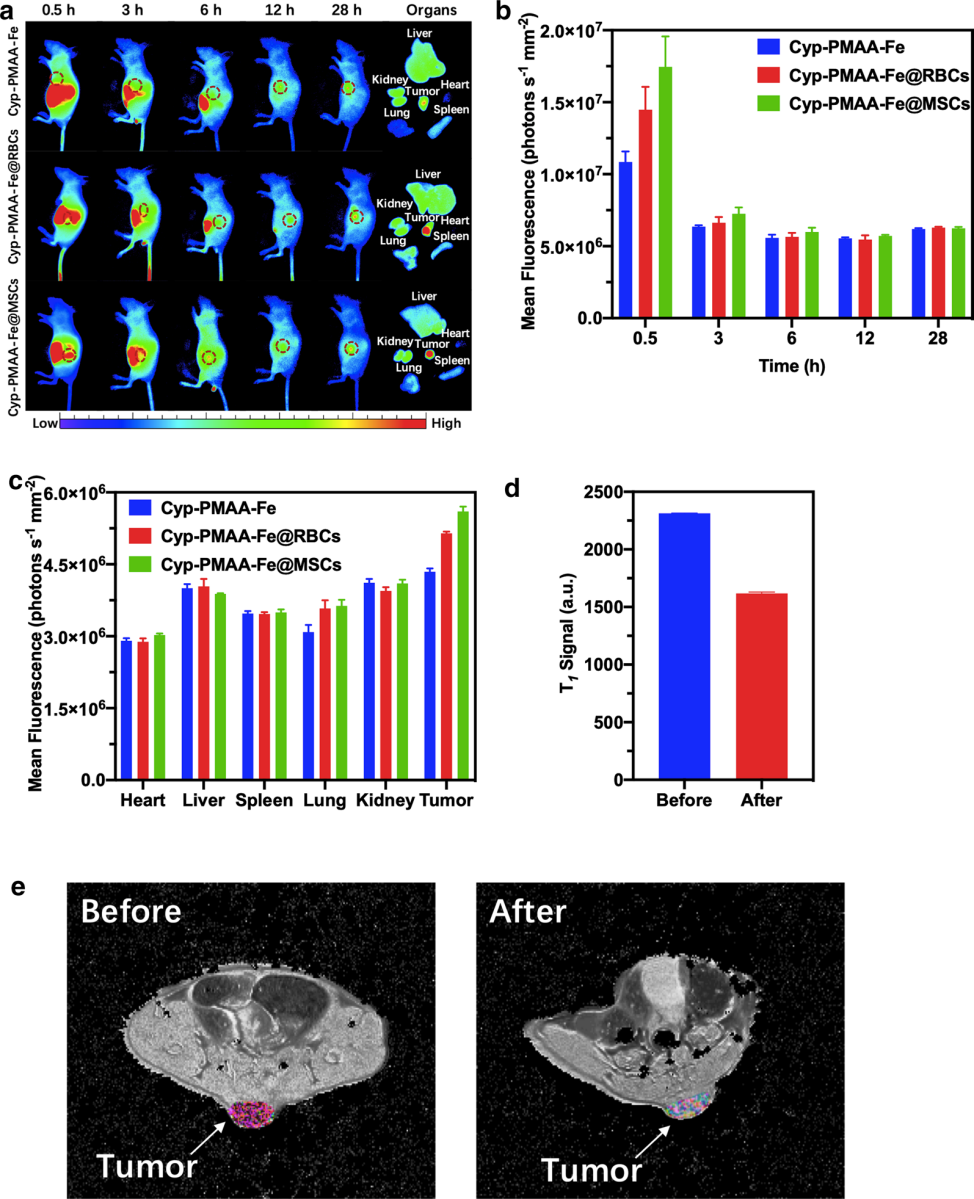
In vivo bimodal fluorescence imaging and *T*_1_-weighted MR imaging. **a** Fluorescence imaging of nude mice injected with Cyp-PMAA-Fe, Cyp-PMAA-Fe@RBCs and Cyp-PMAA-Fe@MSCs. Tumor regions were marked with red circles. **b** Fluorescence intensity data at different time points at the tumor sites. **c** Fluorescence intensity of Cypate in tumors and major organs at 28 h after i.v. injection. **d** *T*_1_-weighted MR signal at tumor sites before and 28 h after i.v. injection of Cyp-PMAA-Fe@MSCs. **e** *T*_1_-weighted MR imaging of LLC1 tumor-bearing mouse before (left) and 28 h after (right) intravenous injection of Cyp-PMAA-Fe@MSCs. Tumor regions were colored

For in vivo fluorescence imaging experiments, the fluorescence signal at the tumor site of the Cyp-PMAA-Fe@MSCs treatment group (n = 3 per group, PMAA = 12 mg kg^−1^) was always stronger than that of the Cyp-PMAA-Fe@RBCs group after intravenous injection into tumor-bearing mice (n = 3 per group), and the difference became the most obvious at 0.5 h under in vivo fluorescence imaging system. The signal intensity at the tumor sites of the Cyp-PMAA@MSCs group was 1.21 times stronger than the Cyp-PMAA-Fe@RBCs group (Fig. 3a, b). Compared with the Cyp-PMAA-Fe@RBCs group, the Cyp-PMAA-Fe@MSCs group showed more nanoparticles enriched at the tumor site, indicating that Cyp-PMAA-Fe@MSCs had better targeting ability than Cyp-PMAA-Fe@RBCs.

For in vivo MR imaging experiments, the *T*_1_ signal decreased 30.01% in 28 h after intravenous injection of Cyp-PMAA-Fe@MSCs (n = 3 per group, PMAA = 12 mg kg^−1^, Fig. 3c, d), which showed that the nanoparticles were accumulated into the tumor sites and promised to apply in *T*_1_-weighted MRI.

**Fig. 4:**
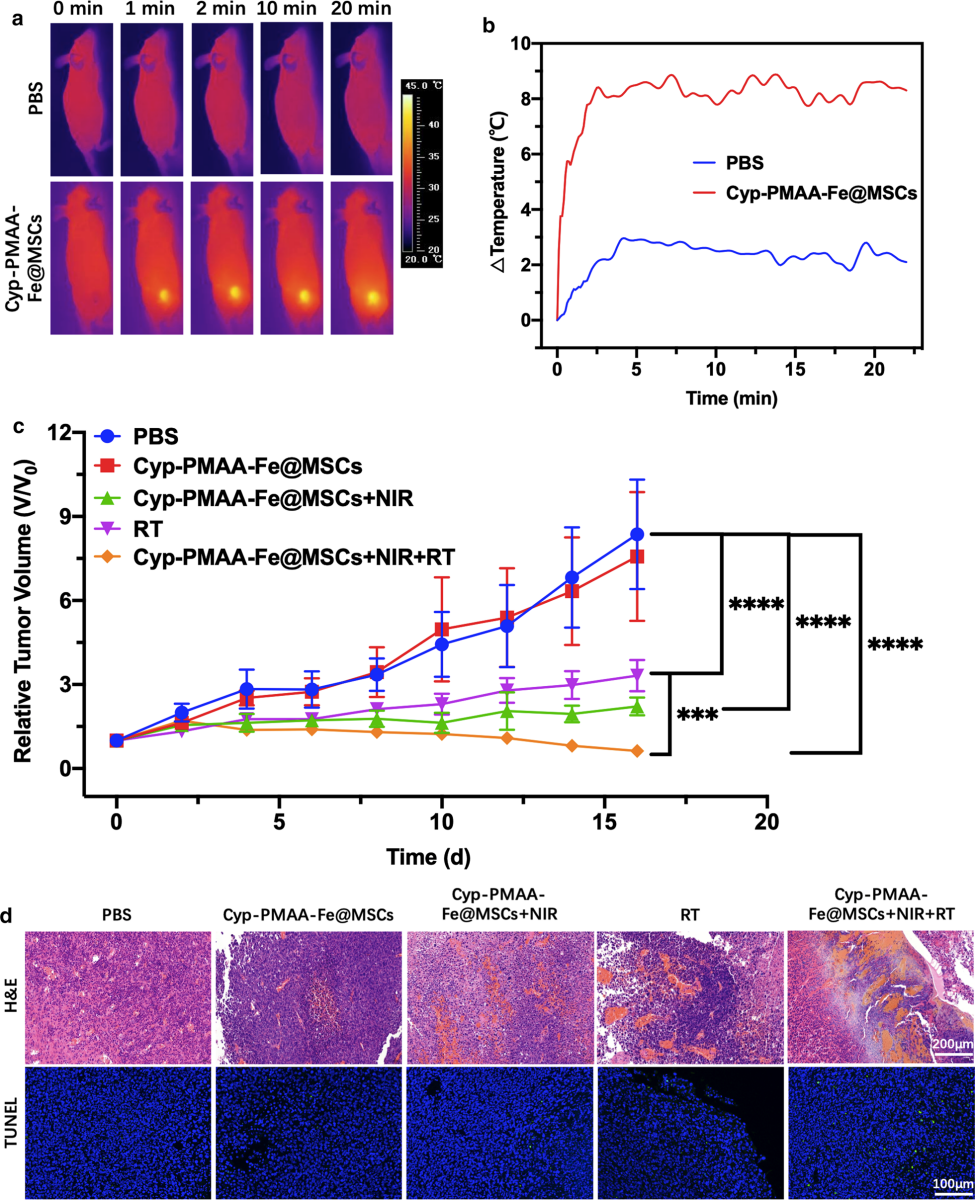
In vivo synergistic combined therapy of LLC1 cancer with Cyp-PMAA-Fe@MSCs. **a** IR images of LLC-1 tumor-bearing mice after i.v. injection of Cyp-PMAA-Fe@MSCs under 808 nm laser irradiation. **b** Temperature changes at tumor sites recorded by IR thermal camera during 808nm laser irradiation. **c** Tumor growth curves of LLC-1 tumor-bearing mice with various treatments. Statistical analysis was performed with Student’s two-tailed t-test (***P<0.005 and ****P<0.001). **d** Images of H&E and TUNEL staining of tumor slices from various groups.

In animal experiments, to evaluate the photothermal effect in vivo, the temperature at the tumor site was measured under NIR irradiation after intravenous injection. In the phosphate buffer saline (PBS) group, the temperature only increased by 1.4 °C after 2 min of irradiation. In 2 min–20 min, the temperature kept fluctuating between 30.2 °C and 31.3 °C. As for the Cyp-PMAA-Fe@MSCs group (n = 3 per group, PMAA = 12 mg kg^−1^), the temperature increased by 9.4 °C within 2 min, and fluctuated between 42 °C and 43 °C in 2 min–20 min (Fig. 4a, b).

To study the in vivo treatment effect, the tumor volume of mice in the PBS group reached 8.36 times larger than the initial value on day 16. The tumor volume of mice injected with Cyp-PMAA-Fe@MSCs (n = 5 per group, PMAA = 12 mg kg^−1^) reached 7.57 times larger than the initial value on the 16th day, indicating that the nanomedicine itself had no inhibitory effect on tumor growth. For Cyp-PMAA-Fe@MSCs + NIR irradiation group (PTT group), the volume of the tumor was 2.22 times larger than the initial value on the 16th day, indicating that the tumor was partially suppressed by PTT. The tumor volume in the RT group was 3.32 times larger than the initial value on day 16 after X-ray irradiation. For Cyp-PMAA-Fe@MSCs + NIR irradiation + RT group (PTT + RT group), the tumor volume on day 16 was only 0.63 times smaller than the initial value, indicating that PTT could enhance RT (Fig. 4c). Hematoxylin–eosin (H&E) staining could provide us with more details in the histopathological level. The PBS group and the group injected with Cyp-PMAA-Fe@MSCs were normal tumor cells with mitotic phases. In the PTT group, there was focal necrosis. Small necrotic foci could be observed in the RT group. As for PTT + RT group, there were a large piece of coagulative necrosis, and balloon-like changes, indicating that PTT combined with RT had a strong killing effect on tumors, resulting in a large number of tumor cells necrosis. Terminal-deoxynucleotidyl transferase mediated nick end labeling (TUNEL) staining characterized the apoptosis and death of cells. The PTT + RT group showed most cell death than any other groups (Fig. 4d). This was consistent with the tumor volume growth curve and the tumor volume test results in animal experiments, which comprehensively proved that using Cyp-PMAA-Fe@MSCs as a nano-PTT agents combined with RT could produce a synergistic effect, substaintially enhancing the RT effect to tumors. The body weight of mice did not increase or decrease significantly. On the 16th day, the body weight of mice in each group was over 90% of the initial value and H&E staining of liver, heart, spleen, lung and kidney of mice in various groups showed no extinct abnormality or damamge (Additional file 1: Figure S7), indicating that there was no obvious malignant consumption in mice, and the physiological condition was acceptable. Cyp-PMAA-Fe@MSCs had no obvious toxic reactions to mice.

## Conclusions

In summary, we successfully developed an intriguing MSCs-engineered nanoplatform to realize multimodal bioimaging and synergestic PTT/RT of NSCLC. By virtue of encapsulation of MSCs on Cyp-PMAA-Fe, Cyp-PMAA-Fe@MSCs nanomedicines were constructed, which exhibited low toxicity, high stability, prominent photothermal-conversion and active-tumor-targeting efficiency. Furthermore, Cyp-PMAA-Fe@MSCs, upon intravenous administration, could accumulate effectively at the tumor sites, which was capable of observation by fluorescence imaging system and *T*_1_-weighted MRI directly, thus enabling desirable PTT-enhanced RT of NSCLC. This work highlights that MSCs-coating nanomedicines are remarkable nanoplatforms to modify nanoagents as biomimetics for active-tumor-targeting. In this regard, it is promising that Cyp-PMAA-Fe@MSCs nanomedicines might be appplicable for multiple bioimaging-guided and photothermal-enhanced RT of NSCLC. It is also conceivable that the novel biomimetic strategy and nanomedicines can be applied for next-generation tumor diagnosis and precise treatment of NSCLC.

## Methods

### Materials and reagents

Methacrylic acid (MAA), divinylbenzene (DVB), 2,2-azobis (isobutyronitrile) (AIBN) were purchased from Shanghai Aladdin Chemistry Co. Ltd., acetonitrile (ACN) and methanol were purchased from Shanghai Lingfeng Chemical Reagent Co. Ltd., ethanol was purchased from Shanghai Zhenxing Chemistry Co. Ltd., iron (III) chloride hexahydrate (FeCl_3_·6H2O), potassium chloride (KCl), magnesium chloride hexahydrate (MgCl_2_·6H_2_O), edetate disodium (EDTANa_2_·2H_2_O), Tris, hydrochloric acid (HCl) and sucrose were all purchased from Sinopharm Chemical Reagent Co. Ltd.. Protease inhibitor, high glucose DMEM medium, fetal bovine serum (FBS), penicillin–streptomycin, phosphate buffer saline (PBS) and trypsin enzyme were purchased from Thermo Fisher Scientific (Waltham, MA, USA). CCK-8 kit was purchased from Dojindo Laboratories (Tokyo, Japan). DAPI, Lyso-tracker green and cell viability (staining of living and dead cells) assay kit were purchased from KeyGen Biotech (Nanjing, Jiangsu, China). Triton X-100 and γ-H2AX antibody were purchased from Sigma-Aldrich (St. Louis, MO, USA). Cypate was synthesized as previously described [46]. Deionized water used in all experiments was obtained from Milli-Q water system.

### Animals

Male nude mice and Balb/c mice (6–8 weeks old) were purchased from Shanghai Slac Lab Animal Ltd. (Shanghai, China) for in vivo studies. Mice were housed in a room with ad libitum access to food and water under a 12 h light/dark cycle. All animal experiments were complied with ethics guidelines of local Ethic Committee and animal laboratories. To establish the LLC-1 tumors, 3 × 10^6^ LLC-1 cells were suspended in serum-free DMEM medium (≈ 50 μL) and injected to the back of each mouse subcutaneously.

### Preparation of PMAA nanoparticles

PMAA nanoparticles were prepared with reflux-precipitation polymerization method. More detailed information was described earlier in ref [44].

### Preparation of PMAA-Fe and Cyp-PMAA-Fe

A typical process for preparing PMAA-Fe was as following: PMAA nanoparticles (25 mg) were dispersed in DI water (6 mL). Excess FeCl_3_·6H_2_O (0.3 mM, 81 mg) were added and the mixture was stirred for 24 h (room temperature, 60 rpm). The product was seperated and purified by repeating centrifugation (8000 rpm for 10 min)/decantation/resuspension for three times. The purified PMAA-Fe was dispersed in ethanol (2 mL) for further use.

Cypate loading process: cypate (5 mg, 20%wt of PMAA) was added to PMAA-Fe/ethanol solution (PMAA = 25 mg). Then, DI water (20 mL) was added to the mixture with ultrasonic bath for 10 min. The product was purified by centrifugation (7000 rpm for 6 min).

### Preparation of MSC membranes, Cyp-PMAA-Fe@MSCs, RBC membranes and Cyp-PMAA-Fe@RBCs

Rat bone marrow MSCs were purchased from Cell Bank of Chinese Academy of Sciences and were identified in our laboratory. MSCs were cultured in high glucose DMEM medium with 10% FBS and 1% penicillin–streptomycin in 37 °C, 5% CO2. The media were changed every 2 days and the cells were passaged by trypsinization before confluence.

MSC membranes were prepared as follows: four 75 cm^2^ cell culture dishes cultured with MSCs (80% confluence) were washed with PBS for 2 times. Then, PBS (6 mL, 4 °C) were added to the dishes. Cells were collected with the cell scraper and centrifugation (4 °C, 500 g, 5 min). Hypotonic lysis (2 mL) containing KCl (10 mM), MgCl_2_(2 mM), Tris–HCl (5 mM, pH = 7.5) and 0.2 tablet of protease inhibitor was added to lyse MSCs. MSCs were pestled by glass homogenizer and centrifuged (4 °C, 3200*g*, 10 min). The supernatant was collected and centrifuged again (4 °C, 20,000*g*, 20 min) to harvest MSC membranes by collecting pellets. Cell conservation (200 μL) consisting of Tris–HCl (10 mM, pH = 7.5) and EDTA (1 mM) was added to preserve MSC membranes at − 80 °C.

MSC membranes enveloping process: Cyp-PMAA-Fe (PMAA = 1 mg) and MSC membrane solutions (10 μL) were mixed in a glass bottle with ultrasonic water bath (4 °C, 2 min). Finally, the solution was stored at 4 °C for other experiments.

The preparation of RBC membranes was reported earlier [47]. The preparation of Cyp-PMAA-Fe@RBCs was similar to the procedure above.

### Characterization

The nanoparticles were characterized with FEI Tecnai F20 TEM. Concentration of Fe was determined by inductively coupled plasma atomic-emission spectroscopy (ICP-AES). The *T*_1_-weighted MRI scanning of Cyp-PMAA-Fe@MSCs was conducted by a 3-T clinical MRI scanner (Discovery MR750, GE, USA).

For protein analysis by SDS-PAGE, the protein concentration of MSC membranes, Cyp-PMAA-Fe and Cyp-PMAA-Fe@MSCs was determined by BCA kit (Pierce, USA). The protein concentration of MSC membranes and Cyp-PMAA-Fe@MSCs was quantified to 1 mg mL^−1^. The PMAA concentration of Cyp-PMAA-Fe and Cyp-PMAA-Fe@MSCs was adjusted to the equal level. All samples were washed by PBS and heated to 70 °C for 10 min and then run in polyacrylamide gel (Beyotime, China) (20 μL for each sample). The gel was stained by Coomassie Brilliant Blue (Beyotime, China) for 1 h and destained in DI water overnight.

The Ultraviolet–visible-NIR spectra were obtained from Ultraviolet–visible-NIR spectrophotometer (PerkinElmer Lambda 750). The loading efficiency and encapsulation rate were determined by the absorbance of cypate at 785 nm. The hydrodynamic diameter and zeta potential of the nanoparticles were measured with a Malvern Zetasizer (Nano ZS90). In photothermal experiment, all the samples (PMAA = 1 mg mL^−1^) were irradiated by an 808 nm continuous-wave laser (Shanghai diffraction Photoelectric Technology Co. Ltd., Shanghai, China). Temperature changes and infrared (IR) images were recorded by an infrared thermal camera (Fotric 225).

### In vitro experiments

The Lewis lung cancer cell line (LLC-1) was purchased from American Type Culture Collection (ATCC, USA) and the culture conditions were the same as MSC.

For the cell viability test, LLC-1 cells were seeded in 96-well plates at a density of 10, 000 cells per well and cultured in an incubator for 24 h. Next, The cells were incubated with PMAA-Fe and Cyp-PMAA-Fe@MSCs of varying concentrations in the incubator for 24 h. The viabilities of those cells were measured by CCK-8 kit.

To evalutae the cellular uptake of Cyp-PMAA-Fe@MSCs, confocal laser scanning microscope (CLSM, ZEISS LSM710, Carl Zeiss, Germany) and flow cytometer (FCM, BD Biosciences) were utilized. PMAA-Fe nanoparticles were loaded with rhodamine B and then enveloped with MSC membranes as the methods described above. 10 000 LLC-1 cells were seeded in each 20 mm glass-bottom culture dish and incubated for 24 h. Next, the cells were incubated with Rho-PMAA-Fe@MSCs (1 mL, PMAA = 200 μg mL^−1^, rhodamine B = 10 μg mL^−1^) for 1 h. Then, the cells were stained with Lyso Tracker Green (1 μL, 100 μM) for 30 min, fixed with 4% polyformaldehyde (1 mL) for 20 min, stained with DAPI (400 μL, 1 μg mL^−1^) and observed under CLSM. For FCM, LLC-1 cells were seeded in 6-well plates at a density of 500 000 cells per well and incubated for 24 h. The cells were incubated with Cyp-PMAA-Fe, Cyp-PMAA-Fe@RBCs and Cyp-PMAA-Fe@MSCs. The cells were measured with FCM at several time points.

To evaluate the photothermal effects of Cyp-PMAA-Fe@MSCs, the Calcein-AM/PI dual staining assay and Cell Counting Kit-8 assay were performed. For dual staining assay, 150 000 LLC-1 cells were seeded in each 20 mm glass-bottom culture dish and incubated for 24 h. The cells were then incubated with PMAA-Fe@MSCs/Cyp-PMAA-Fe@MSCs (PMAA = 200 μg mL^−1^) for 2 h. The cells were then irradiated with 808 nm laser (power = 1.5 W cm^−2^) for 5 min and put in the incubator for 2 h. Then, the cells were stained with calcein-AM (2 μM) and PI (8 μM) for 30 min and observed with CLSM. For photothermal viability assay, LLC-1 cells were seeded in 96-well plates at a density of 10, 000 cells per well and incubated for 24 h. The following incubation and irradiation methods were the same as dual staining assay, cell viabilities were measured with CCK-8 kit.

For γ-H2AX immunofluorescence analysis, LLC-1 cells were seeded in 12-well plates at a density of 10 000 cells per well and incubated for 24 h. The cells were incubated with Cyp-PMAA-Fe@MSCs (PMAA = 200 μg mL^−1^) for 6 h and then irradiated with X-ray (4 Gy). The cells were incubated for 2 h, fixed by 4% paraformaldehyde (1 mL) for 10 min, incubated with 0.2% Triton X-100 for 10 min, incubated with 1% BSA for 1 h and incubated with the primary antibody (mouse monoclonal anti-phospho-histone γ-H2AX, 1% BSA in PBS, 1:500) overnight at 4 ℃. The cells were incubated with the secondary antibody (sheep anti-mouse Cy633, 1% BSA in PBS, 1:500) for 1 h, stained with DAPI (400 μL, 1 μg mL^−1^) for 5 min and observed with CLSM.

For clonogenic assay, LLC-1 cells were seeded in 6-well plates at a density of 100, 200, 300, 500, and 1 000 cells per well and incubated for 24 h. The cells were incubated with PMAA-Fe@MSCs/Cyp-PMAA-Fe@MSCs (PMAA = 200 μg mL^−1^) for 6 h and then irradiated with X-ray (0, 2, 4, 6, and 8 Gy). The cells were incubated for 10 days, fixed with anhydrous ethanol and stained with Crystal violet (CV, Sigma-Aldrich). The surviving fraction was determined by the final colonies.

### In vivo bimodal imaging

For fluorescence imaging, 6 tumor-bearing mice were randomly divided into two groups (n = 3 per group, tumor volume ≈ 200 mm^3^) and intravenously injected with Cyp-PMAA-Fe/Cyp-PMAA-Fe@MSCs (150 μL, PMAA = 2 mg mL^−1^). The mice were captured at various time points with the Optical and X-ray small imaging system (Bruker, excitation: 780 nm, emission: 845 nm). The mice were sacrificed at 28 h post-injection and their tumors as well as major organs were captured as well.

For MR imaging, 3 tumor-bearing mice (tumor volume ≈ 200 mm^3^) were intravenously injected with Cyp-PMAA-Fe@MSCs (150 μL, PMAA = 2 mg mL^−1^). The mice were scanned by 7 T small animal MRI (BRUER, BioSpec 70/20, USA) before and 28 h post-injection of Cyp-PMAA-Fe@MSCs, respectively.

### In vivo cancer therapy

30 tumor-bearing mice were randomly divided into five groups (n = 5 per group, tumor volume ≈ 70 mm^3^) for various treatment: (I) PBS, (II) Cyp-PMAA-Fe@MSCs, (III) Cyp-PMAA-Fe@MSCs + NIR, (IV) RT, (V) Cyp-PMAA-Fe@MSCs + NIR + RT. The mice were intravenously injected with Cyp-PMAA-Fe@MSCs (150 μL, PMAA = 2 mg mL^−1^). PTT was conducted 28 h after i.v. injection, with 808 nm laser (power = 1.5 W cm^−2^) for 20 min. The temperature at the tumor sites was recorded by IR thermal camera and the temperature was kept between 42.5 °C and 43.5 °C. RT was conducted after PTT, at a dose of 8 Gy. The size of the tumors was measured by Vernier calipers every two days, and the tumor volume was calculated by V = *a* × *b*^2^/2, where *a* and *b* were the longest and shortest diameter axes of the tumor, respectively. The body weight of the mice was measured at the same time as an indicator for systemic toxicity. The mice were sacrificed and the tumors were collected for H&E staining as well as TUNEL staining at day 16.

### Statistical analysis

The data were presented as the mean ± SD. Statistical analysis was performed with GraphPad Prism (version 8.0). For comparisons, any two groups were compared using the two-tailed unpaired Student's t- test. Comparisons between more than two groups were conducted using one-way analysis of variance procedures. Differences were considered statistically significant when p < 0.05.

## Supplementary Information

**Additional file 1: Figure S1.** Structural formula of cypate. **Figure S2.** Hydrodynamic diameters of PMAA-Fe and Cyp-PMAA-Fe@MSCs and zeta potenials of PMAA-Fe, MSCs and Cyp-PMAA-Fe@MSCs. **Figure S3.** UV–vis-NIR spectra and typical photos of cypate with various concentrations. **Figure S4.** Absorbance of cypate with various concentrations at 785 nm. **Figure S5.** IR images of Cyp-PMAA-Fe@MSCs at 808 nm laser with various powers. **Figure S6.** Heating and cooling curve of Cyp-PMAA-Fe@MSCs. **a** The temperature change of Cyp-PMAA-Fe@MSCs response to 808 nm laser on and off in period of 1800s. **b** Linear regression of time versus –lnθ obtained from the cooling period of NIR laser off. **Figure S7.** H&E staining of tissue slices from several organs in various groups.

## Data Availability

All data generated or analyzed during this study are included in this published article.
