# Supplementary material for: MSCs-engineered biomimetic PMAA nanomedicines for multiple bioimaging-guided and photothermal-enhanced radiotherapy of NSCLC
Source: J Nanobiotechnology. 2021 Mar 20;19:80. doi: 10.1186/s12951-021-00823-6 (PMC7981797; doi:10.1186/s12951-021-00823-6)
Supplement: Supplementary file 1 — Additional file 1: Figure S1. Structural formula of cypate. Figure S2. Hydrodynamic diameters of PMAA-Fe and Cyp-PMAA-Fe@MSCs and zeta potenials of PMAA-Fe, MSCs and Cyp-PMAA-Fe@MSCs. Figure S3. UV–vis-NIR spectra and typical photos of cypate with various concentrations. Figure S4. Absorbance of cypate with various concentrations at 785 nm. Figure S5. IR images of Cyp-PMAA-Fe@MSCs at 808 nm laser with various powers. Figure S6. Heating and cooling curve of Cyp-PMAA-Fe@MSCs. a The temperature change of Cyp-PMAA-Fe@MSCs response to 808 nm laser on and off in period of 1800s. b Linear regression of time versus –lnθ obtained from the cooling period of NIR laser off. Figure S7. H&E staining of tissue slices from several organs in various groups. [file 12951_2021_823_MOESM1_ESM.docx]

**Supplementary Information**

MSCs-Engineered Biomimetic PMAA Nanomedicines for Multiple Bioimaging-Guided and Photothermal-Enhanced Radiotherapy of NSCLC

Yipengchen Yin,^‡^ Yongjing Li,^‡^ Sheng Wang,^‡^ Ziliang Dong, Chao Liang, Jiaxin Sun, Changchun Wang, Rong Chai, Weiwei Fei, Jianping Zhang, Ming Qi, Liangzhu Feng, Qin Zhang^*^

Department of Radiation Oncology, Shanghai Chest Hospital, Shanghai Jiao Tong University, Shanghai 200030, P. R. China;

^‡^These authors contributed equally to this work.

^*^Corresponding author.

^*^Qin Zhang: Email: zhangqin@shsmu.edu.cn (Q. Zhang)

**
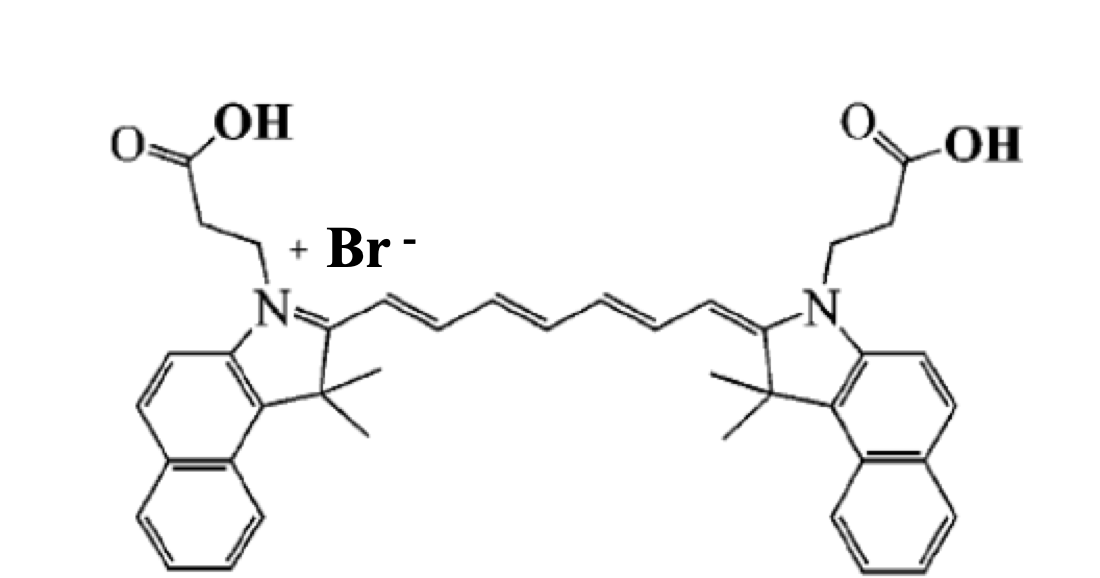
**

**Figure S1.** Structural formula of cypate.

**
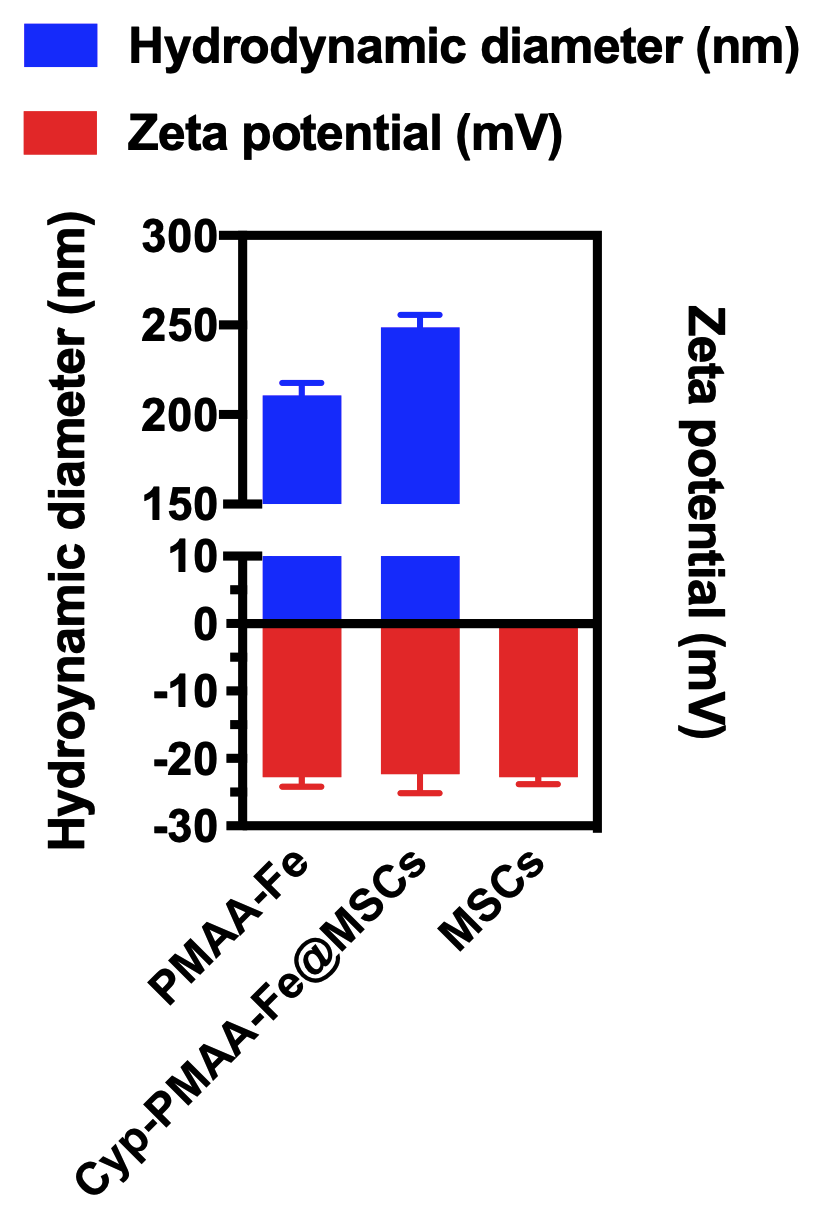
**

**Figure S2.** Hydrodynamic diameters of PMAA-Fe and Cyp-PMAA-Fe@MSCs and zeta potenials of PMAA-Fe, MSCs and Cyp-PMAA-Fe@MSCs.

**
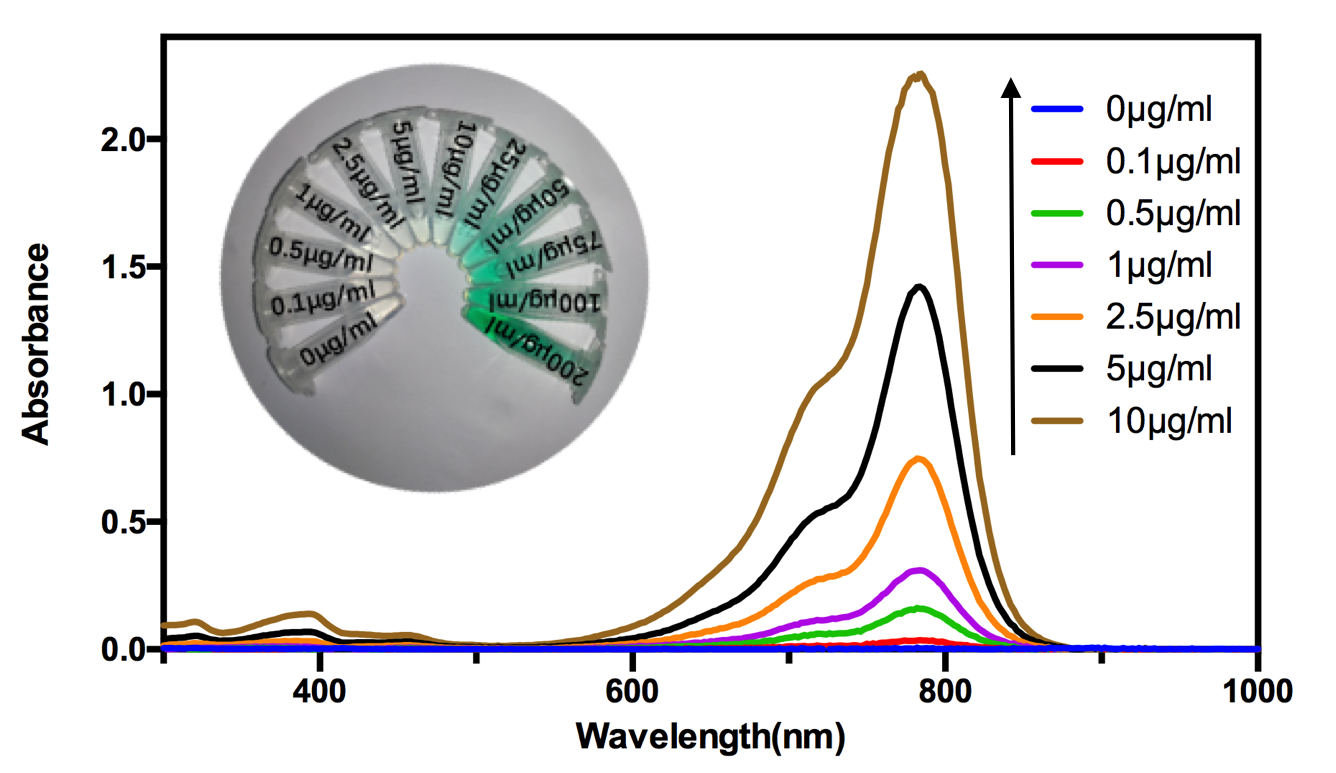
**

**Figure S3.** UV-vis-NIR spectra and typical photos of cypate with various concentrations.


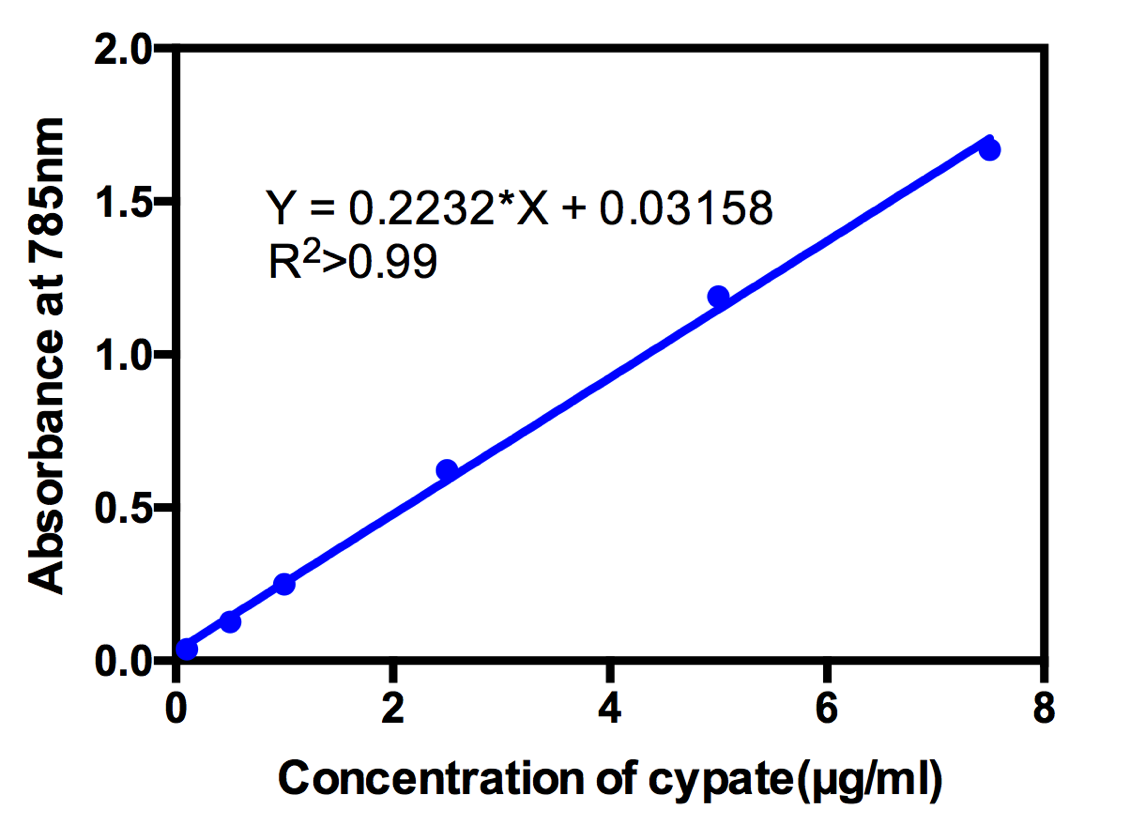


**Figure S4.** Absorbance of cypate with various concentrations at 785 nm.


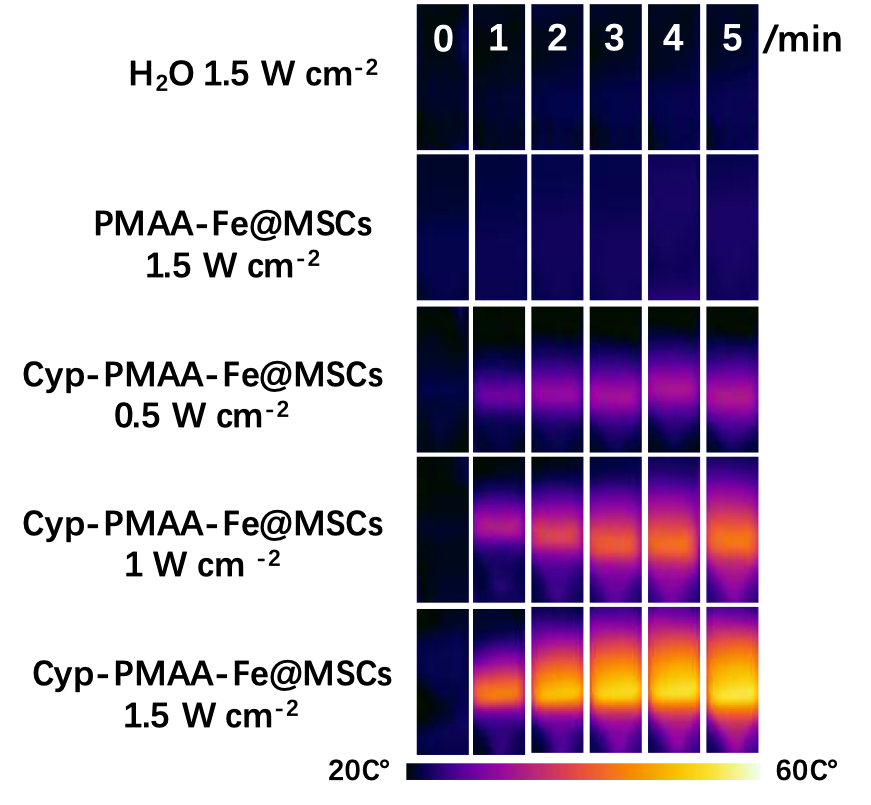


**Figure S5.** IR images of Cyp-PMAA-Fe@MSCs at 808 nm laser with various powers.


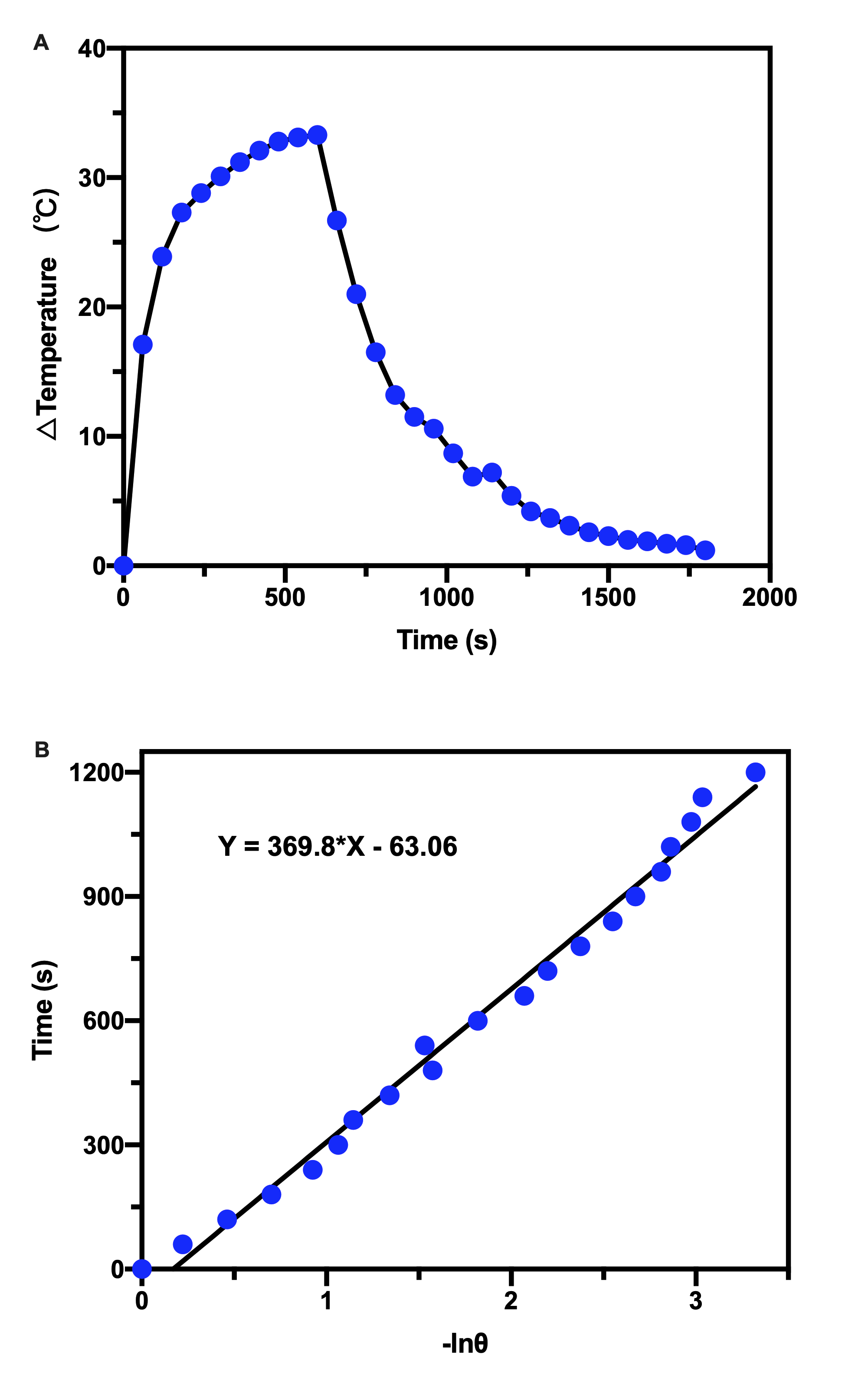


**Figure S6.** Heating and cooling curve of Cyp-PMAA-Fe@MSCs. (A) The temperature change of Cyp-PMAA-Fe@MSCs response to 808 nm laser on and off in period of 1800 s. (B) Linear regression of time versus –lnθ obtained from the cooling period of NIR laser off.


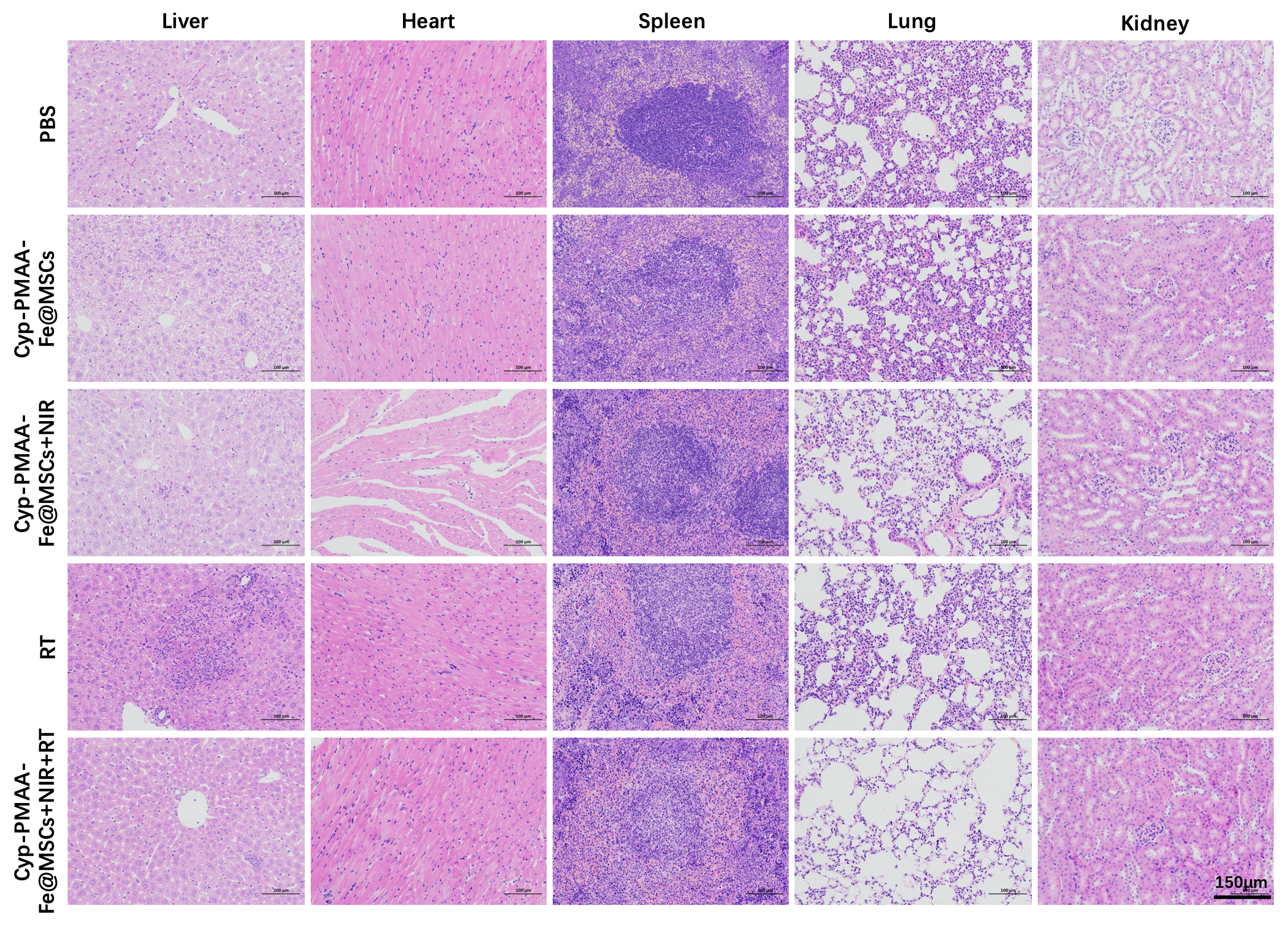


**Figure S7.** H&E staining of tissue slices from several organs in various groups.
